# Treatment of Pneumonia in Childhood

**Published:** 1891-03

**Authors:** L. P. Barber

**Affiliations:** Tracy City, Tennessee


### TREATMENT OF PNEUMONIA IN CHILDHOOD.

BY L. P. BARBER, M. D., TRACY CITY, TENNESSEE.

Pneumonia is a specific, self-limited disease, with no known specific remedy. Its treatment will vary with varying patients —will be symptomatic. Being a self-limited disease, the wise physician will be careful not to give needless medication. A previously healthy and strong child with a non-complicated case will require little medicine, and none should be given except as indicated, unless indeed we are forced to administer some harmless potion as a sedative to the parental brain. The indications for medicines I meet as follows. When first called we frequently find signs of gastro-hepatic congestion, such as a flabby tongue with whitish fur, anorexia, and often considerable nausea. A few small doses of calomel followed by a saline laxative will then be of service. Usually at my first visit I order a tepid bath or sponging, and this is to be continued several times daily so long as the fever lasts. Sometimes following the bath I have the patient annointed with some bland oil in which quinine is mixed; these inunctions add much to the little invalid's comfort. Pain is the most common indication for treatment, and for it we have one principal remedy—opium. Opium, however, should be used very, very carefully in the quite young. Liquid Dover's Powder, the camphorated tincture of opium, or the deodorized tincture, are the best forms for children. It should be used until it relieves the pain, but not to produce much somnolence. Opium is also indicated when the patient is very nervous—nerve on a rack—half a drop of the deodorized tincture of opium every two hours to a child one year old will ease the pain, quiet the nervousness, and often avert convulsions. This remedy is also of great benefit in quieting the sometimes almost constant cough. A prescription like the following has been helpful to me for specially harrassing cough combined with a state of high nervous tension:

| | | |
|---|---|---|
| ℞ | Tr. opii devd. - - - - | gtt. vj. x. |
| | Tr. hyoscyami - - - - | ʒ j |
| | Syr. ipecac - - - - | ʒ j-jss |
| | Syr. bal. tolut. - - - - | ℥ ss |
| | Aquae minth. ad. - - - - | ℥ ij |

M. Sig.—One teaspoonful every two or three hours to a child one year old.

After the attack has reached its height, and especially if there seem much depression, I leave the ipecac out of the prescription and give muriate of ammonia as:

℞

| | | |
|---|---|---|
| Tr. opii devd. | - - - - | gtt. vj-x |
| Ammon. muriat | - - - - | gr. xij-xx iv |
| Syr. bal. tolut. | - - - - | ℥ ss |
| Aquae ad. | - - - - | ℥ ij |

M. Sig.—One teaspoonful every two to three hours.

If there are evidences of heart failure, and symptoms of exhaustion appear as the case advances, I use the carbonate instead of the muriate of ammonia. I make more frequent use of ammonia, the carbonate and muriate, than of any other medicines. I often use one or the other from the beginning to the end of an attack. For the depression alcohol is also indicated. Good rye whisky is my favorite form. A weakly child may need it from the beginning. But here let me condemn the routine use of alcohol in this disease. Many cases will not need it, and when not needed alcohol like all other medicines should not be used. The temperature of pneumonia is often high, but I do not believe it is an element of special danger. It will seldom or never need more treatment than sponging and the tepid bath. Do not use anti-pyrine or anti-febrine to reduce the fever in pneumonia of infancy. In cases where it is necessary to avoid the use of opium, small doses of these medicines might be given to relieve the pain and quiet the restlessness. But these heart depressants should be used very carefully. Of this class of medicines phenacetine is safest and should be preferred for children. Quinine should not be used as an antipyretic. In a few adult cases, early in the attack, where the area of inflammation seemed rapidly extending, I have thought it was arrested by giving large doses of quinine, and perhaps it would prove of benefit in children under similar circumstances. It should be administered per rectum, the watery solution of the muriate or bi-sulphate being used. During convalescense I use small doses of quinine as a tonic, administering it in the syrup of licorice.

In malarial districts, and in all asthenic cases, these small doses of quinine may be profitably used from the beginning. Many find an indication for aconite or veratrum viride in the earlier stages of this disease. Cardiac failure we all agree is the great source of danger, and I cannot reconcile the inconsistency of adding the depressing effects of a drug to the pathological influence that is already weakening that organ. The advocates of veratrum talk about "bleeding the patient into his own veins." To me it seems more rational to bleed into a basin, then we relieve the heart without embarrassing it with a drug effect. Bleeding is sometimes indicated in pneumonia, but very rarely in the case of a child. Digitalis is sometimes needed to sustain a failing heart, but it should not be used indiscriminately. The patient should have food at frequent intervals; during the first few days he will take but little at a time. A nursing child should be given water frequently, and the breast should be allowed every three hours. Weaned children should be given milk, and pepsin may be used to assist digestion. Water should be given freely to a patient of any age. A plaster composed of one part ground mustard to three parts of linseed meal should be applied early to the inflamed area. Leeches are often of help. Hot poultices are much used, but I doubt their utility. A jacket of cotton batting answers every purpose of the poultice, and has not its disadvantages. The child should not be allowed to remain in one position too long. It should be in a bed or crib most of the time, and not held in the arms of the nurse. See that it is comfortably clothed in soft flannel, but not kept too warm. The extremities should be watched to see that they are always warm. The room in which the patient is should be large, well lighted and well ventilated. If possible, it should have an open fire-place, as this secures the best ventilation, and fresh, pure air is of prime importance to the patient with crippled lungs. Finally, we should not forget that rest and quiet are just as important to our small patients as to those of larger growth.

